# The mitochondrial ATP synthase is a shared drug target for aging and dementia

**DOI:** 10.1111/acel.12715

**Published:** 2018-01-07

**Authors:** Joshua Goldberg, Antonio Currais, Marguerite Prior, Wolfgang Fischer, Chandramouli Chiruta, Eric Ratliff, Daniel Daugherty, Richard Dargusch, Kim Finley, Pau B. Esparza‐Moltó, José M. Cuezva, Pamela Maher, Michael Petrascheck, David Schubert

**Affiliations:** ^1^ Cellular Neurobiology The Salk Institute for Biological Studies La Jolla CA USA; ^2^ Donald P. Shiley BioScience Center San Diego State University San Diego CA USA; ^3^ Centro de Biología Molecular CIBERER, Universidad Autónoma de Madrid Madrid Spain; ^4^ Chemical Physiology The Scripps Research Institute La Jolla CA USA

**Keywords:** aging, Alzheimer's disease, ATP synthase, dementia, metabolism, mitochondria, neurodegeneration

## Abstract

Aging is a major driving force underlying dementia, such as that caused by Alzheimer's disease (AD). While the idea of targeting aging as a therapeutic strategy is not new, it remains unclear how closely aging and age‐associated diseases are coupled at the molecular level. Here, we discover a novel molecular link between aging and dementia through the identification of the molecular target for the AD drug candidate J147. J147 was developed using a series of phenotypic screening assays mimicking disease toxicities associated with the aging brain. We have previously demonstrated the therapeutic efficacy of J147 in several mouse models of AD. Here, we identify the mitochondrial α‐F_1_‐ATP synthase (ATP5A) as a target for J147. By targeting ATP synthase, J147 causes an increase in intracellular calcium leading to sustained calcium/calmodulin‐dependent protein kinase kinase β (CAMKK2)‐dependent activation of the AMPK/mTOR pathway, a canonical longevity mechanism. Accordingly, modulation of mitochondrial processes by J147 prevents age‐associated drift of the hippocampal transcriptome and plasma metabolome in mice and extends lifespan in drosophila. Our results link aging and age‐associated dementia through ATP synthase, a molecular drug target that can potentially be exploited for the suppression of both. These findings demonstrate that novel screens for new AD drug candidates identify compounds that act on established aging pathways, suggesting an unexpectedly close molecular relationship between the two.

## 1 INTRODUCTION

Although great efforts toward AD drug discovery have been made in recent years, there is currently an impasse with only one new AD therapeutic approved since 2004 (Cummings, Morstorf, & Zhong, 2014). While most AD drugs are the result of target‐based screens, a lack of credible drug targets apart from amyloid‐β and tau has limited the search. Phenotypic screens provide an alternative drug discovery strategy that does not require a priori knowledge of a target. Because age is the greatest risk factor for AD, we developed a unique phenotypic screening paradigm specifically designed to recapitulate several of the most common age‐associated central nervous system (CNS) toxicities using cell culture models (Prior et al., 2014). We identified a synthetic compound called J147 that is neuroprotective in all of these assays (Chen et al., 2011) and promotes the division of neuronal precursor cells in vivo and in vitro (Prior et al., 2016). Behaviorally, it enhances memory and restores cognition in APPswe/PS1ΔE9 and the rapidly aging senescence‐accelerated mouse prone (SAMP8) dementia mouse models (Currais et al., 2015; Prior, Dargusch, Ehren, Chiruta, & Schubert, 2013; Morley, Armbrecht, Farr, & Kumar, 2015). Here, we identify the mitochondrial α‐F1 subunit of ATP synthase (ATP5A) as a high affinity molecular target of J147, a protein previously studied in the context of aging (Chin et al., 2014). Therefore, our analysis not only identifies a new AD drug target but also causally connects metabolic regulation, aging, and dementia through a single molecular drug target.

## 2 RESULTS

### 2.1 Target identification

**Figure 1:**
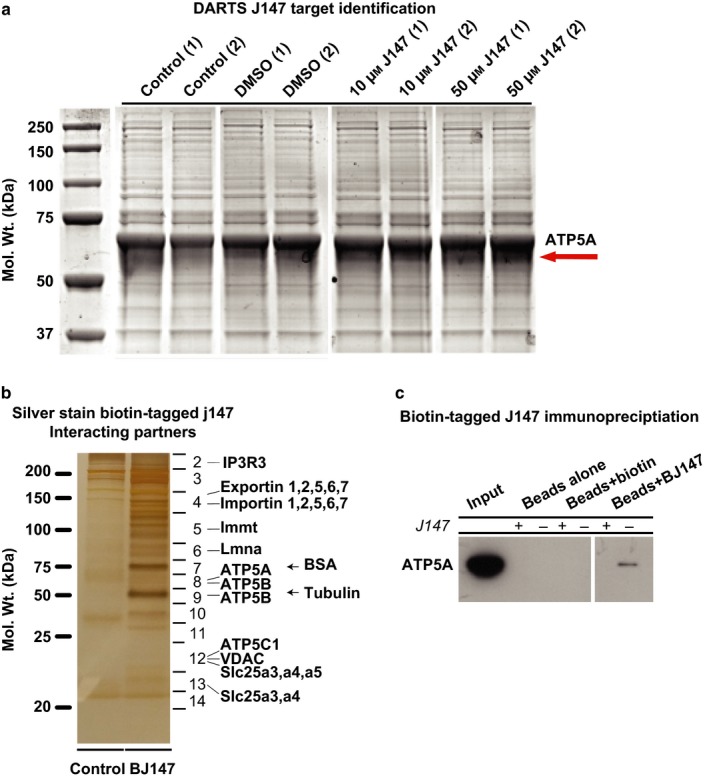
Target identification by DARTS and affinity precipitation pull‐downs. (a) DARTS revealed ATP5A as a putative direct J147 target. HT22 cells were treated with 10 and 50 μm J147 for 15 min after which cells were lysed using M‐PER and digested with Pronase. The only band visually preserved among J147‐treated samples (10 and 50 μm) (red arrow) was excised and analyzed by LC/MS/MS along with control lanes. ATP5A was the most enriched protein indicating direct target engagement. (b) Affinity precipitation with a biotinylated derivative of J147 (BJ147) pulled down an enriched fraction of mitochondrial‐associated proteins. (c) Affinity precipitation using subventricular zone (SVZ) lysates from adult mice demonstrates BJ147 binding to ATP5A. Unlabeled J147 (100 μm) outcompeted ATP5A binding to BJ147

Three independent approaches were used to initially identify and then confirm the molecular target of J147. First, we used an unbiased small molecule target identification approach called drug affinity responsive target stability (DARTS) (Lomenick, Jung, Wohlschlegel, & Huang, 2011) to detect putative binding partners. Lysates from the HT22 hippocampal nerve cell line were incubated with vehicle, 10 or 50 μm J147 for 15 min before treating with pronase to degrade unbound protein complexes. A band containing proteins preserved by J147 treatment following protein electrophoresis was identified, and mass spectrometry (MS) analysis determined that ATP5A was the most enriched putative target relative to controls (Figure 1a, red arrow, Figure S1). ATP5A is a catalytic subunit of the mitochondrial ATP synthase complex responsible for the synthesis/hydrolysis of ATP.

To confirm ATP5A as the molecular target of J147, we performed several additional experiments. First, we incubated HT22 cells and mouse subventricular zone (SVZ) tissue lysates with a biotinylated derivative of J147, BJ147, and used LC/MS/MS to identify coprecipitating proteins. Consistent with the DARTS experiment, a strong enrichment of mitochondria‐associated proteins was present in the streptavidin pull‐down fraction from the BJ47‐incubated samples. The only protein that was reproducibly identified in both the pull‐down and DARTS experiments was ATP5A, while other mitochondrial proteins involved in ion flux and transport such as inositol 1,4,5‐triphosphate receptor 3 (IP3R3), members of the solute carrier family 25 (SLC25a3‐5), and voltage‐dependent anion channel (VDAC) were only present in the pull‐down (Figure 1b). Importantly, the amount of ATP5A was greatly reduced in BJ147‐precipitated lysates incubated with excess unlabeled J147 as a binding competitor (Figure 1c).

**Figure 2:**
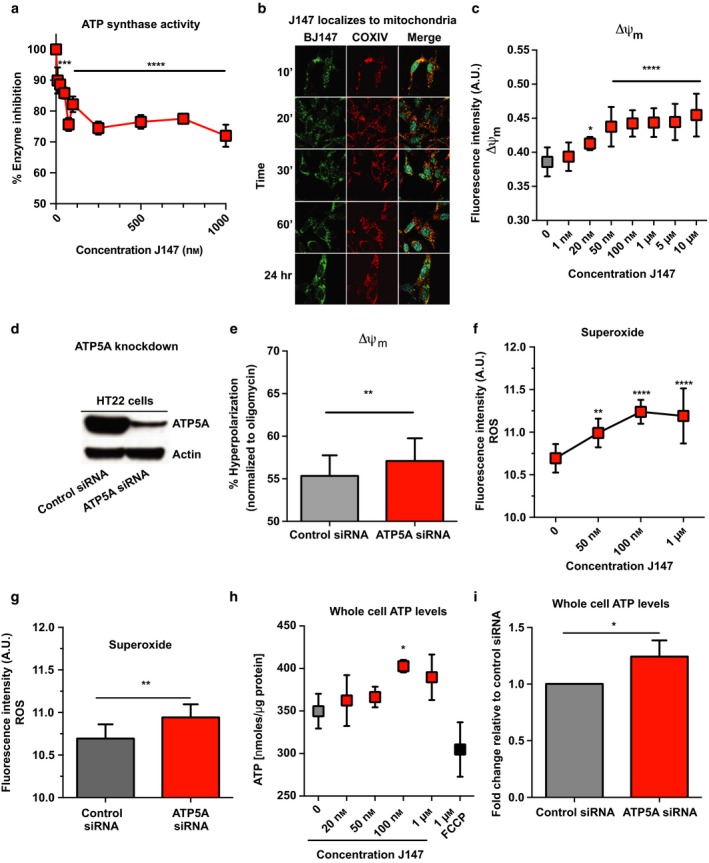
J147 targets mitochondrial bioenergetics. (a) J147 inhibition of ATP synthase activity from isolated bovine heart mitochondria (*F*(9,20) = 40.72, ***p = .0004, ****p < .0001, ANOVA). Oligomycin, an inhibitor of both forward (ATP synthesis) and reverse (ATP hydrolysis) ATP synthase activity and an inactive derivative of J147, CAD120, served as positive and negative controls, respectively (not shown) (CAD120: 97.1%; oligomycin: 14 ± 2.4%). (b) BJ147 (green) localizes to mitochondria (red) in HT22 cells within 10 min of addition to tissue culture media. DAPI (cyan), nuclei, BJ147 (green), COXIV (red). Scale bar = 10 μm. (c) Dose‐dependent increase in mitochondrial membrane potential (Δψ_m_) in HT22 cells following J147 treatment. Statistics reported for J147 treatments compared to vehicle (*p = .0483, ****p < .0001, ANOVA). (d) ATP5A knockdown in HT22 neuronal cells. (e) ATP5A knockdown phenocopies the effect of J147 on Δψ_m_ (**p = .001, *t* test, *t* = 3.385, df = 94). JC1 fluorescence (aggregate:monomer) normalized to oligomycin (10 μm). (f) J147 dose‐dependently increases mitochondrial superoxide production in HT22 neuronal cells. (g) siRNA knockdown of ATP5A revealed a similar increase in mitochondrial superoxide production (**p = .001, *t* test, control siRNA compared to ATP5A siRNA). (h) J147 increases whole cell ATP levels in HT22 cells. FCCP served as a negative control (*p = .0374, ANOVA). (i) ATP5A siRNA‐targeted knockdown increased whole cell ATP levels (*p = .0326)

The most highly enriched protein in both the DARTS and affinity precipitation experiments was ATP5A. Therefore, we asked whether the activity of the ATP synthase complex is modulated by J147. First, we tested J147's effect on ATP synthase enzyme kinetics in isolated bovine heart mitochondria. A dose–response curve is shown after 1 hr of J147 incubation (Figure 2a) that indicates saturating partial inhibition (23.6 ± 3.4%) of ATP synthase activity by J147 with an EC_50_ of 20 nm. Importantly, this inhibition is only partial, even at saturating concentrations of J147. These results demonstrate that J147 binds to and partially inhibits the activity of the mitochondrial ATP synthase.

We next determined whether the intracellular localization of J147 in HT22 cells was consistent with a mitochondrial target by confocal fluorescent microscopy using BJ147. An imaging time course demonstrated that J147 colocalized with the mitochondrial marker cytochrome C oxidase IV (COXIV) (Figure 2b, Table S1). Localization of J147 was rapid, occurring within 10 min. Thus, both biochemical and localization experiments support ATP5A as a target of J147.

### 2.2 J147 targets mitochondrial bioenergetics

ATP synthase couples the production or hydrolysis of ATP to the transport of H^+^ ions across the inner mitochondrial membrane, making it a direct regulator of mitochondrial polarity (Δψ_m_). We tested J147's effect on Δψ_m_ using JC1, a ratiometric cationic dye. A significant dose‐dependent increase in mitochondrial membrane potential is observed within 1 hr of J147 treatment (Figure 2c), an effect consistent with the regulation of ATP synthase activity (Perry, Norman, Barbieri, Brown, & Gelbard, 2011). To independently confirm that targeting the α‐subunit of ATP synthase affects its activity and thus Δψ_m_, we performed siRNA‐targeted knockdown of ATP5A (Figure 2d) and found a similar increase in Δψ_m_ when taken as percent oligomycin‐induced hyperpolarization (Figure 2e). ATP5A siRNA‐treated cells displayed reduced capacity for hyperpolarization after oligomycin addition.

Changes in ATP synthase activity occur concomitantly with the production of reactive oxygen species (ROS) (Laura Formentini, Sánchez‐Aragó, Sánchez‐Cenizo, & Cuezva, 2012). Although traditionally thought of as being detrimental, new evidence suggests that inhibition of ATP synthase can elicit a retrograde, ROS‐mediated prosurvival response (Laura Formentini et al., 2012). Both J147 treatment and ATP5A knockdown caused a significant increase in superoxide levels within the mitochondria (Figure 2f,g).

In the elderly and in patients with AD, mitochondrial dysfunction leads to reduced levels of ATP which may contribute to disease progression (Reddy et al., 2012; Zhang, Rissman, & Fend, 2015). J147 increased ATP levels in HT22 cells within 4‐6 hr of treatment (Figure 2h) without affecting the rate of glycolysis (Figure S2). Furthermore, ATP5A siRNA‐targeted knockdown similarly increased whole cell ATP levels in these cells (Figure 2i) without affecting protein levels or composition of other oxidative phosphorylation (oxphos) components (Figures S3 andS4).

### 2.3 ATP synthase inhibition protects from neurotoxic insults

**Figure 3:**
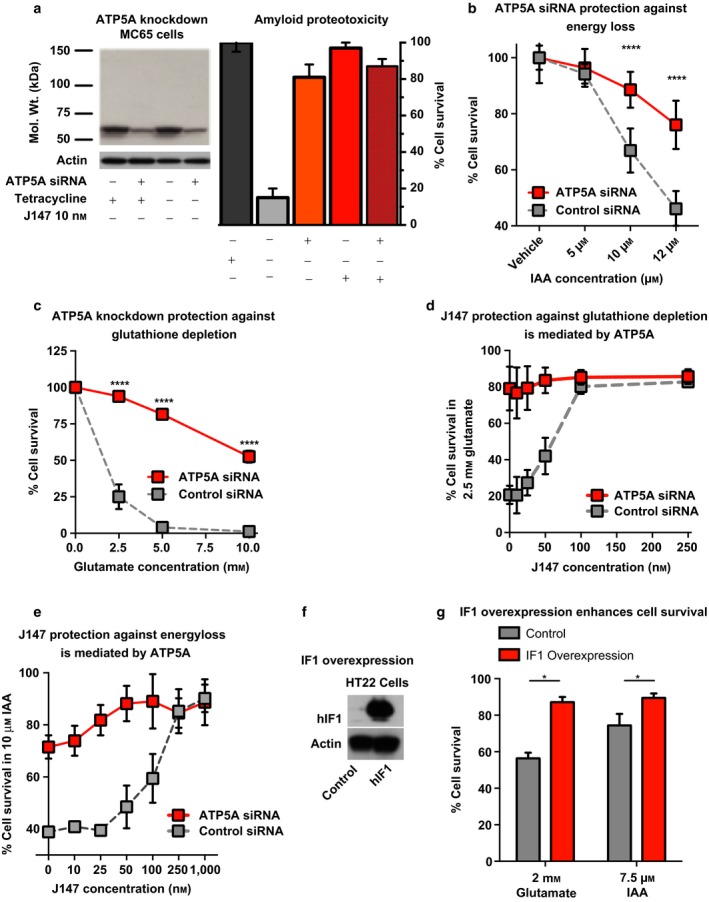
Knockdown of ATP5A phenocopies the neuroprotective effects of J147. (a) ATP5A knockdown efficiency in MC65 cells (left, Western blot). Both J147 and ATP5A knockdown protect MC65 cells from death in a proteotoxicity model of Aβ (right). (b) ATP5A knockdown protects HT22 cells from cell death in a model of IAA‐induced energy depletion (****p < .0001, *t* test). (c) ATP5A knockdown protects HT22 cells from cell death in a model of glutamate‐induced oxytosis (*p* = .0002, ****p < .0001, *t* test). (d, e) ATP5A knockdown does not provide an additive effect to J147‐induced protection during oxytosis and IAA toxicity. (f, g) Overexpression of IF1 is neuroprotective against glutamate (2 mm) and IAA‐induced energy depletion (7.5 μm) in HT22 cells (*p = .0180, *t* test)

We next asked whether inhibiting ATP synthase activity elicits a similar neuroprotective response as seen with J147 in our age‐associated toxicity screens that were the basis for J147 development (Prior et al., 2013). If so, modulating ATP synthase activity either by siRNA‐targeted knockdown of ATP5A or overexpression of its endogenous inhibitor, ATPase inhibitor factor 1 (IF1), should protect in models of amyloid proteotoxicity, glutamate‐induced glutathione depletion (oxytosis), and iodoacetic acid (IAA)‐induced energy depletion. First, we tested protection against amyloid proteotoxicity using human MC65 neuronal cells conditionally expressing the C99 fragment of amyloid precursor protein (APP) under the control of a tetracycline (tet) promoter. Upon induction, C99 is processed to produce Aβ polymers and this leads to cell death, an effect that is blocked by J147 (Chen et al., 2011). Similarly, ATP5A knockdown also prevented intracellular amyloid‐induced cell death in MC65 cells (Figure 3a).

Next, we tested the neuroprotective effects of ATP5A knockdown in toxicity models of oxytosis and energy depletion. Oxytosis occurs when high levels of extracellular glutamate block cystine import resulting in glutathione depletion and cell death (Tan, Schubert, & Maher, 2001), while IAA irreversibly inhibits the glycolytic enzyme glyceraldehyde 3‐phosphate dehydrogenase to induce energy loss (Maher, Salgado, Zivin, & Lapchak, 2007). We have previously shown that J147 protects HT22 cells from both oxytosis and IAA toxicity (Chen et al., 2011). Knockdown of ATP5A phenocopies the protection conferred by J147 in both assays (Figure 3b,c). As expected if the target of J147 is ATP5A, cell viability is not further improved by J147 treatment in ATP5A siRNA‐targeted knockdown cells (Figure 3a,d,e).

To corroborate the neuroprotection induced by ATP5A knockdown, we overexpressed a constitutively active, pH‐insensitive mutant (H49K) of the endogenous inhibitor of ATP synthase, IF1 (Figure 3f) in HT22 cells. IF1 binds to the catalytic F1‐portion of ATP synthase and inhibits its activity (García‐bermúdez & Cuezva, 2016). Similar to J147 and ATP5A knockdown, IF1‐overexpression significantly protected HT22 cells from glutamate and IAA‐induced toxicity (Figure 3g).

Together, these data demonstrate that modulating ATP synthase activity, whether by siRNA‐mediated knockdown of ATP5A or IF1 overexpression, phenocopies the neuroprotective effects of J147 in aging‐associated and AD‐like toxicities, and further support ATP5A as the molecular target for J147.

### 2.4 J147 and ATP5A modulate AMPK/mTOR signaling

As age is the greatest risk factor for AD, interventions that slow aging or extend health span might serve as potential therapies that delay disease onset (Currais, 2015). Recent studies have highlighted a role for ATP synthase in the regulation of mTOR and lifespan extension in flies and worms (Chin et al., 2014; Sun et al., 2014). Inhibition of mTOR via activation of AMPK is a canonical longevity‐associated pathway (Johnson, Rabinovitch, & Kaeberlein, 2013). Activation of AMPK is achieved through phosphorylation of threonine (Thr) 172 on the α‐subunit, lowering activity of some ATP‐consuming pathways while promoting ATP synthesis through others such as fatty acid oxidation. Therefore, we asked whether J147 modulated AMPK/mTOR signaling via ATP synthase.

**Figure 4:**
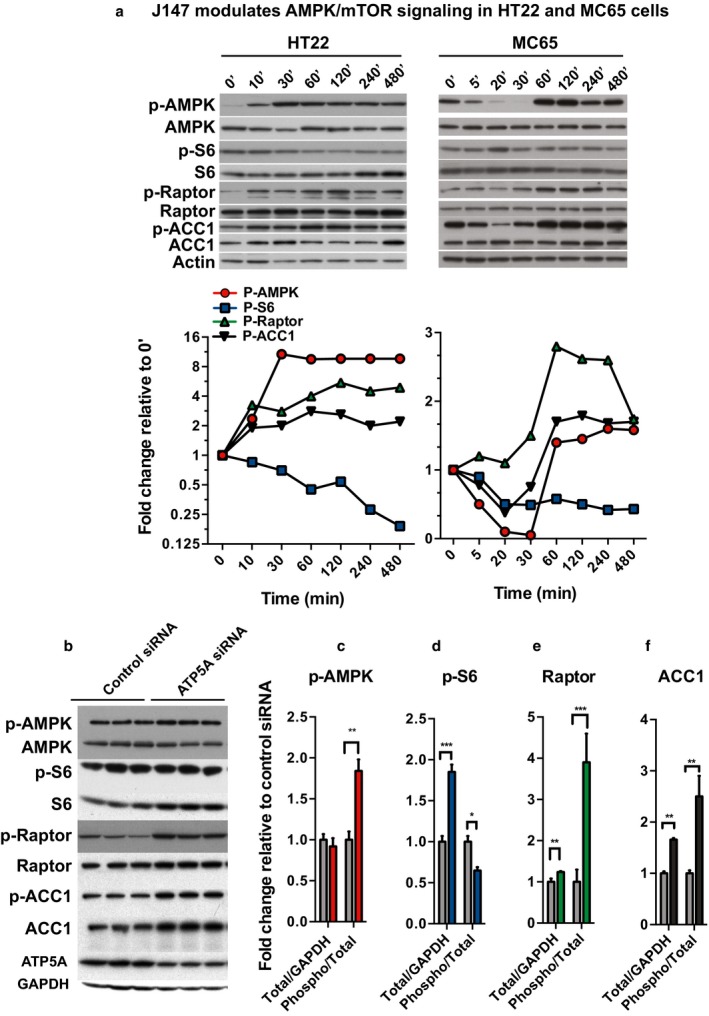
J147 and ATP5A modulate AMPK/mTOR signaling. (a) Time course of 100 nm J147 activation of the AMPK/mTOR signaling pathway. Increasing phosphorylation of AMPK (α‐Thr172), raptor (Ser792), ACC1 (Ser79) and decreasing phosphorylation of S6 (Ser235/236) in HT22 and MC65 cells. Corresponding quantification graphs for AMPK/mTOR targets are below their respective Western blots. (b) ATP5A knockdown in MC65 cells phenocopies the J147 effect on the AMPK/mTOR pathway. Corresponding quantifications for each target are shown (c–e). Grey columns represent control siRNA, and all other colors represent ATP5A siRNA. (c) Increases in phosphorylation of AMPK (red) (**p = .0011 (phospho/total); (d) decrease in phosphorylation of S6 (blue) (Ser235/236) (**p = .0017 (phospho/total), ***p = .0002 (tot/GAPDH); (e) increase in phosphorylation of raptor (green) (Ser792) (**p = .0027 (phospho/total), **p = .0073 (tot/GAPDH); (f) increase in phosphorylation of ACC1 (black) (Ser79) (**p = .003 (phosphor/total), ****p < .0001 (tot/GAPDH)

We monitored AMPK/mTOR activity using site‐specific phosphorylation antibodies against proteins involved in this pathway. In Figure 4a, two different cell types were used to assay the activity of the AMPK/mTOR pathway following treatment with J147. In mouse HT22 (left panel) and human MC65 neuronal cells (right panel), there is a clear, time‐dependent activation of AMPK (pAMPK) by J147. AMPK phosphorylation of raptor at Ser792 is critical for AMPK‐mediated inhibition of mTOR. An increase in raptor Ser792 phosphorylation was observed in both cell types treated with J147 (Figure 4a). Raptor‐mediated inhibition of mTORC1 activity reduces unnecessary ATP expenditure by decreasing S6 kinase activity resulting in reduced protein translation. AMPK‐mediated phosphorylation of acetyl‐CoA carboxylase (ACC1) promotes energy production by reducing fatty acid synthesis and increasing β‐oxidation of fatty acids (Hardie, Ross, & Hawley, 2012). J147 decreases S6 activity and increases ACC1 phosphorylation in both cell types. These data show that the AMPK/mTOR signaling pathway, known to promote aging, is downstream of J147 target engagement. Importantly, siRNA‐mediated knockdown of ATP5A in MC65 cells (Figure 4b) phenocopied the effects of J147 on AMPK/mTOR signaling (Figure 4b‐f).

**Figure 5:**
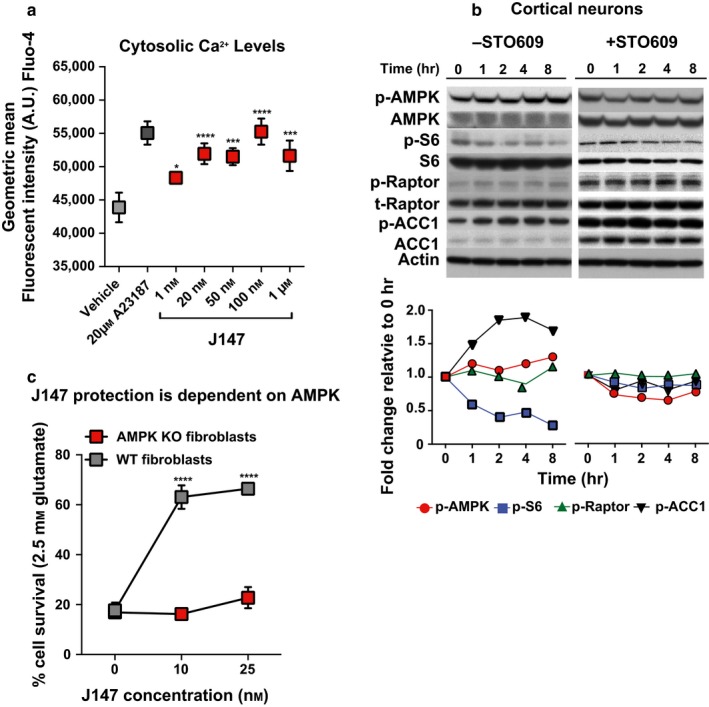
J147 modulates resting Ca^2+^ homeostasis to activate the AMPK/mTOR axis. (a) J147 increases the levels of cytosolic Ca^2+^ in HT22 cells. A23187 was used as a positive control (*p = .0162, ***p = .0002, ***p < .0001, ANOVA). (b) The CamKK2 inhibitor STO‐609 attenuated J147‐induced activation (100 nm) of AMPK targets in rat primary cortical neurons. Phosphorylation targets include AMPK (α‐Thr172), Raptor (Ser792), ACC1 (Ser79), and S6 (Ser235/236); corresponding quantifications for each are shown below. (c) J147 protection against glutamate (2.5 mm) is reduced in AMPK KO fibroblasts demonstrating a direct role for AMPK in the protective effects of J147 (****p < . 0001 (10 nm J147),****p < . 0001 (25 nm J147),***p < .0001 (100 nm J147))

J147 caused an increase in AMPK phosphorylation despite modestly increasing ATP levels, suggesting an alternative mode of AMPK activation to that of sensing the AMP:ATP ratio. The only known alternative in the brain is calcium/calmodulin‐dependent protein kinase kinase β (CamKK2) activation of AMPK (Racioppi & Means, 2012), suggesting Ca^2+^‐mediated activation of AMPK by J147. Therefore, we asked whether J147 might regulate CamKK2 activity by modulating resting Ca^2+^ homeostasis. Measurement of cytosolic Ca^2+^ upon J147 treatment in HT22 cells revealed a dose‐dependent increase in cytosolic Ca^2+^ levels (Figure 5a). To determine whether CamKK2 mediates J147 activation of AMPK, we treated rat cortical neurons with J147 and a potent inhibitor of CamKK2, STO‐609. STO‐609 prevented J147‐mediated activation of AMPK and its downstream signaling effectors, ACC1, S6, and raptor (Figure 5b), thereby identifying CamKK2 as key mediator of the modulation of AMPK/mTOR signaling by J147 (statistics shown for 30 min and 4 hr time points in Figure S5). An AMPK knockout (KO) fibroblast cell line was used to further examine the role of AMPK in J147 signaling. We found a significant decrease in J147‐mediated protection by J147 in the oxytosis assay in the KO cells as compared to WT cells (Figure 5c).

### 2.5 J147 attenuates age‐associated decline and extends lifespan

**Figure 6:**
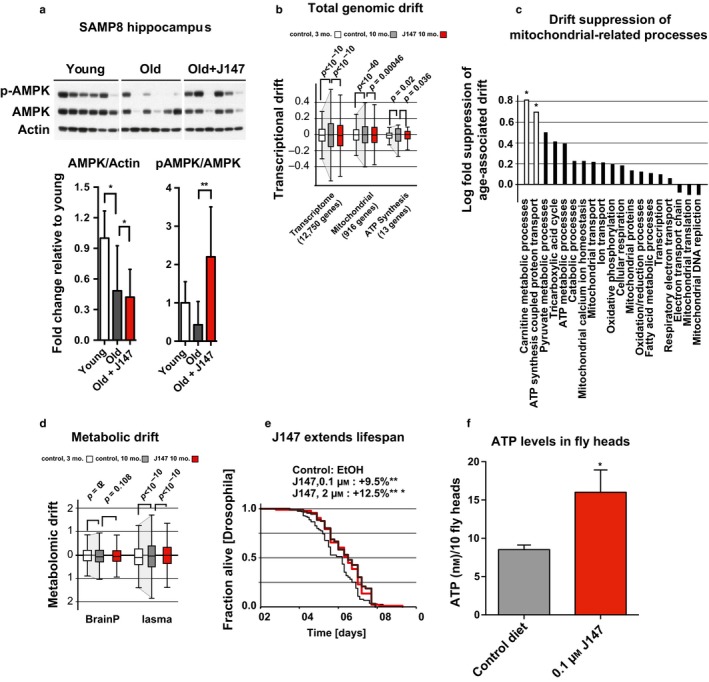
J147 attenuates age‐associated decline and extends lifespan in vivo. (a) Old (10 months) male SAMP8 mice treated with J147 exhibit increased phosphorylation of AMPK in hippocampal lysates similar to the levels in young (3 months) SAMP8 mice. Corresponding bar graphs are shown beneath the Western blots (Tukey's multiple comparison), AMPK/actin (*p = .0453 (young vs. old)*,* and *p = .0241 (young vs. *J147)),* pAMPK/AMPK (**p = .0093 (old vs. J147). (b) J147 suppresses age‐associated hippocampal transcriptional drift. *p*‐values displayed on graph (Tukey box plot). (c) Specific drift suppression of processes involving carnitine metabolism and ATP synthesis‐coupled proton transport were significant. (d) Significant suppression of metabolomic drift was observed in plasma metabolites; *p*‐values displayed on graph (Tukey box plot). (e) 0.1 and 2 μm J147 increase longevity in male Drosophila by 9.5% (red line) and 12.5% (brown line), respectively (*F*(2, 386) = 7.654, *p = .0118, ***p = .0009, ANOVA *)*. (f) 0.1 μm J147 increases ATP levels in drosophila heads (feeding started at 1 week) (*p = .0011, ANOVA)

Our results thus far demonstrate that J147 protects from age‐associated brain toxicities in cell culture models via interaction with ATP synthase and subsequent modulation of the AMPK/mTOR pathway. We therefore asked whether AMPK signaling is activated by J147 in SAMP8 mice, an accelerated aging and sporadic dementia model, and whether the J147‐induced physiological changes in these mice are consistent with delayed aging. Hippocampi from mice fed J147 for 6 months, starting at 3 months of age, were isolated and used for Western blot analysis of AMPK (Currais et al., 2015). J147 significantly increased the phosphorylation of AMPK in the old SAMP8 mice, so that the phosphorylation more closely resembled that of young mice (Figure 6a).

As J147 appears to target a canonical aging pathway, we next asked whether J147 treatment delayed signatures of aging. Recently, it has been shown that aging progressively destabilizes the transcriptome, resulting in a drift in mRNA transcript levels away from those observed in young animals (Rangaraju et al., 2015). As some longevity mechanisms dramatically suppress age‐associated transcriptional drift and preserve a youthful transcriptome phenotype (B Rangaraju et al., 2015), we asked whether J147 had a similar effect. Analysis of hippocampal gene expression data from 3‐ and 10‐month‐old SAMP8 mice confirmed that transcriptional drift variance increased with age across the entire transcriptome (Figure 6b). However, treatment with J147 attenuated age‐associated transcriptional drift, reducing its variance by ~6% (*p* < 10^−10^) and preserving a more youthful transcriptional profile. Interestingly, the expression of *IF1*, the endogenous inhibitor of ATP synthase, was significantly downregulated in aged SAMP8 mice (young/old fold change = 0.18; *p < .05), suggesting that a loss in the regulated control of ATP synthase activity occurs during the aging process.

Because J147 targets mitochondrial ATP synthase, we then asked whether J147 specifically suppressed the age‐associated drift of gene transcripts involved in mitochondrial functions. Indeed, J147 dramatically suppressed transcriptional drift of transcripts encoding mitochondrial components of both carnitine metabolism and ATP synthesis‐coupled proton transport (Figure 6c). Carnitine is critical for maintaining mitochondrial function and ATP synthesis as it is required for the transport of long chain fatty acids into the mitochondria, resulting in their oxidation and production of acetyl‐CoA for entry into the tricarboxylic acid cycle (TCA cycle) (Bratic & Larsson, 2013). We have previously shown that J147 reduces the age‐associated accumulation of acylcarnitine in old SAMP8 mice that manifest AD‐like pathology (Currais et al., 2015). This suggests that modulating ATP synthase activity may restrain the age‐associated loss of orchestrated gene expression involved in coordinating mitochondrial bioenergetics.

Similar to assessing transcriptional drift, metabolic drift analyses can be used to monitor the aging of the metabolome. Because mitochondria are essential for maintaining metabolic homeostasis, we asked whether J147 had a protective effect on metabolic drift similar to its effect on the transcriptome. We performed targeted metabolomics on brain and plasma extracts from 3‐ and 10‐month‐old SAMP8 control or J147‐treated mice (Currais et al., 2015). No significant differences in the drift of the brain metabolome were observed between old and young animals. However, we did detect a substantial metabolomic drift in plasma metabolites, an effect that was attenuated by J147 treatment (Figure 6d). Together, these analyses demonstrate that J147 treatment stabilizes the hippocampal transcriptome and plasma metabolome against age‐associated drift observed in SAMP8 mice.

Due to its effect on AMPK/mTOR signaling and suppression of transcriptional/metabolic drift, we then asked whether J147 could extend lifespan using Drosophila. In these experiments, 100 nm and 2 mm J147 administered in the food starting at 1 week of age increased longevity, extending median lifespan by 9.5% and 12.8%, respectively (Figures 6e and S6). In addition, the effects of J147 on ATP levels observed in vitro were recapitulated in vivo (Figure 6f), where a significant increase in ATP levels in Drosophila heads was detected in a short‐term feeding paradigm of 100 nm J147 for 48 hr.

## 3 DISCUSSION

In this study, we show that J147, an AD drug candidate identified by phenotypic screening that has shown benefits in animal models of AD, acts via the mitochondrial protein ATP5A, a subunit of ATP synthase, to promote cell survival and reduce specific changes associated with aging. The identification of a drug target affecting aging as well as age‐associated cognitive decline strongly suggests that aging and dementia are closely related on a molecular level, perhaps more so than previously thought (Bredesen, 2014).

Mitochondria regulate a variety of metabolic signaling pathways and are involved in programs of cell survival and death (Galluzzi, Kepp, & Kroemer, 2012). They are uniquely poised to integrate calcium signals with energy metabolism (Martínez‐Reyes & Cuezva, 2014). Within the past two decades, mounting evidence has suggested a causal relationship between mitochondrial dysfunction and AD (Picone, Nuzzo, Caruana, Scafidi, & Di Carlo, 2014). Therefore, proper metabolic control is critical for mounting a successful response to the toxic stresses afflicting the aging brain and may provide alternatives to the amyloid pathway for AD‐targeted therapeutic interventions.

While our kinetic data demonstrating ~20% inhibition of ATP synthase activity suggest an allosteric regulation by J147, it does not exclude involvement of other protein binding partners. Interestingly, the mitochondrial‐associated proteins VDAC, Slc25a, and IP3R3 were also identified in BJ147‐pull‐down samples from HT22 cells and SVZ tissue. These proteins (Baines, 2009), along with ATP synthase (Bernardi, Rasola, Forte, & Lippe, 2015), have been implicated in the composition of the mitochondria permeability transition (mPT) pore responsible for executing cell death programs during lethal conditions of stress via mitochondrial Ca^2+^ efflux. This has led to the idea of targeting the mPT pore as a potential therapy for neurodegeneration (Rao, Carlson, & Yan, 2014). However, the only protein shared between the DARTS and pull‐down experiments was ATP5A, and its knockdown prevented cell death in the oxytosis, IAA‐and Aβ‐induced toxicity models used as screening assays for the development of J147. These data suggest that ATP5A is the primary target mediating J147's effects against neurotoxicity.

Recently, a specific in vivo role for ATP synthase inhibition in the protection of brain neurons against excitotoxic damage was demonstrated through the generation of a conditional mouse model expressing the human form of mutant ATPase inhibitory factor 1 (hIF1) which leads to sustained inhibition of ATP synthase (L Formentini et al., 2014). Our results demonstrating an increase in IF1‐mediated protection in our toxicity assays corroborate the neuroprotective effect seen with ATP5A siRNA‐mediated knockdown and support the idea that ATP synthase inhibition is neuroprotective.

The observation that J147 increased cytosolic Ca^2+^ levels likely explains the activation of CamKK2 and led us to investigate the role of CamKK2 in AMPK phosphorylation. Ca^2+^/CaM‐mediated activation of CamKK2 leads to AMPK activation in the brain (Racioppi & Means, 2012). Inhibition of CamKK2 with STO609 abolished the effects of J147 on AMPK activation. Although it is not clear how inhibition or knockdown of ATP5A leads to compartmental changes in Ca^2+^ levels, it is not surprising given that Ca^2+^ flux from the mitochondria relies on H^+^ pumping and concomitant regulation of Δψ_m_ by ATP synthase (Brookes, Yoon, Robotham, Anders, & Sheu, 2004). Importantly, AMPK was required to mediate J147 protection, again indicating a direct role for AMPK in J147's mechanistic pathway. Furthermore, ATP5A siRNA‐mediated knockdown not only phenocopies the neuroprotective effects of J147 in vitro, but also recapitulates the increase in ROS, Δψ_m,_ and ATP in HT22 neuronal cells.

AMPK is considered to be an energy sensor inhibiting anabolic processes that consume energy and promoting catabolic processes that produce energy (Inoki, Kim, & Guan, 2012). Therefore, we asked whether the aforementioned pathways were affected both in vitro and in vivo by J147 treatment. In cell culture, J147 activated AMPK/mTOR signaling via increased phosphorylation of the downstream target ACC1. Importantly, modulation of ATP synthase activity via siRNA‐targeted knockdown of ATP5A phenocopied the J147 effect on AMPK/mTOR targets. Furthermore, Western blot analysis on hippocampal lysates from J147‐treated SAMP8 mice indicated that AMPK is activated in vivo. Collectively, these data argue that J147‐mediated neuroprotection elicited by targeting ATP synthase may regulate both metabolism and aging.

As recent studies have demonstrated a role for reduced ATP synthase activity in promoting lifespan extension in worms and flies via inhibition of mTOR signaling (Chin et al., 2014; Sun et al., 2014), we asked whether J147 could extend lifespan in drosophila. In these experiments, J147 extended median lifespan up to 12.5%. As lifespan extension studies are much more difficult to perform in mice, we next asked whether J147 affects the aging phenotype in SAMP8 mice using a recently developed transcriptional drift analysis that detects age‐associated changes at the molecular level (Rangaraju et al., 2015). We examined hippocampal gene expression and targeted metabolomic data of J147‐treated and untreated old SAMP8 mice. J147 treatment from a young age stabilized the hippocampal transcriptome as well as the plasma metabolome against age‐associated increases in drift variance suggesting a biologically younger transcriptome and metabolome. Stabilization was most profound on processes associated with mitochondrial metabolism. Because J147 was identified based on its ability to protect cells from old age‐associated neurotoxicities in vitro, these results strongly imply that aging and age‐associated dementia are much more closely related than previously assumed and may share common drug targets. If the close relationship between preventing aging and dementia observed for J147 holds true for other genetic targets identified in aging research, these pathways would provide a new source of AD drug targets that are desperately needed.

## 4 EXPERIMENTAL PROCEDURES

### 4.1 Cell lines

Mouse hippocampal HT22 and human MC65 neuronal cells were propagated as previously described (Davis & Maher, 1994; Sopher, Fukuchi, Kavanagh, Furlong, & Martin, 1996). MC65 cells were a generous gift of Dr. Sopher Primary cortical neurons were prepared from embryonic day 17 Sprague‐Dawley rats as described (Chen et al., 2011). Induction of intracellular amyloid toxicity in MC65 human neuronal cells (human) was performed as described previously (Chen et al., 2011). AMPK knockout (K.O.) fibroblasts were from Ruben Shaw (Salk Institute).

### 4.2 Cell viability and acute toxicity

Cell viability was determined by MTT assays in 96‐well plates (Davis & Maher, 1994). Oxytosis, iodoacetic acid (IAA), and Aβ toxicity assays were performed as previously reported (Prior et al., 2014).

### 4.3 Transcriptome/metabolome drift analysis

Senescence‐accelerated mouse prone 8 (SAMP8) mice were acquired from Harlan Laboratories (U.K.) and used as previously described for whole transcriptome and metabolomics analysis (Currais et al., 2015). Young (3 months) and old (10 months) male SAMP8 mice were fed with either control diet or chow containing J147 (~10 mg/kg per day) (Currais et al., 2012). Transcriptional drift analysis was performed as previously described (Rangaraju et al., 2015) with the exception that we removed expressed genes below the 20th percentile. Experiments were performed in accordance with the US Public Health Service Guide for the Care and Use of Laboratory Animals and protocols approved by the Salk Institute IACUC.

### 4.4 Darts

HT22 cells were treated with 10 and 50 μm J147 15 min. Cells were lysed using M‐PER (Pierce, 78503) with the addition of protease inhibitors and phosphatase inhibitors (Roche, 11697498001 and 4906845001). Lysates were cleared at 14,000 RPM for 15 min, adjusted with M‐PER to equivalent protein levels and digested with Pronase (Roche, 10165921001) for 10 min at room temperature. The digests were separated by SDS‐PAGE and visualized by Coomassie blue staining. Unique bands in J147‐treated samples (representing putative protein targets spared from proteolysis) as compared to matched control lanes were excised, trypsin‐digested, and subjected to MS, then searched using Scaffold™ Proteome Software 2.0. Significant identifications were required to have at least two peptides with Sequest X_corr_ values of 2.0.

### 4.5 J147 pull‐downs

Biotin‐J147 was used in pull‐down experiments. HT22 cells and adult male mice subventricular zone brain samples were lysed in lysis buffer (20 mm HEPES, 50 mm KCl, 20 mm MgCl_2_, 20 mm Na_2_MoO_4_, 0.1% NP40) on ice. Lysates were precleared with streptavidin magnetic beads (Pierce, 88816), followed by incubation at 4°C overnight with 10 μm J147 or Biotin‐J147 with and without J147 (100 μm). The following day, the bead‐J147 complexes were washed, eluted with sample buffer, and run on an SDS‐PAGE gel. Whole lanes for each condition were cut into 12 pieces and the proteins from each submitted for protein identification.

### 4.6 Immunofluorescent staining

HT22 cells were plated on glass coverslips in 24‐well plates. The following day, cells were treated with 20 μm Biotin‐J147 (BJ147) for various times. Cells were immunostained as previously described (Prior et al., 2016) using anti‐COXIV (1:500, Cell Signaling, 4844).

### 4.7 Complex V activity assay

Complex V activity was assayed using the MitoTox OXPHOS Complex V Activity Kit (Abcam, ab109907) per the manufacturer's instructions. An inactive derivative of J147, CAD120 (100 nm) and DMSO were used as negative controls and oligomycin (10 μm) (Sigma, 75351) as a positive control.

### 4.8 Western blot

Western blots were performed as previously described (Currais et al., 2014). Antibodies used were as follows: APP C‐terminal (Sigma, A8717), ATP5A (Abcam, 14748), Total Oxphos Rodent antibody cocktail (ab110413), pAMPK (Cell Signaling, 2535), AMPK (Cell Signaling, 2793), pS6 (Cell Signaling, 4858), S6 (Cell Signaling, 2317), pRaptor (Cell Signaling, 2083), raptor (Cell Signaling, 2280), pACC1 (Cell Signaling, 11818), ACC1 (Cell Signaling, 4190), and Actin (BD Transduction Laboratories™, 4125). Horseradish peroxidase‐conjugated secondary antibodies: goat anti‐rabbit, goat anti‐mouse (1:5,000, Bio‐Rad, 1706516, 1721019). The CamKK2 inhibitor STO‐609 (Cayman 15325) was used at 1 μg/ml. NativePAGE Western blots were performed according to manufacturer's protocol (ThermoFisher Scientific, BN1001).

### 4.9 ROS and membrane potential measurements

Superoxide (Molecular Probes, MitoSox M36008) and mitochondrial membrane polarity (Molecular Probes, JC‐1 T3168) experiments were performed according to the manufacturer's instructions. J147 was added to cells for at least 1 hr before the addition of dyes. JC1 (1 μg/ml) and MitoSox (2.5 μm) were added to cells for 45 and 15 min, respectively at 37°C. Fluorescent measurements were immediately read on a Spectramax M5 plate reader (Molecular Devices). JC‐1 monomer and aggregate fluorescence were measured independently. Oligomycin (10 μm) and FCCP (10 μm) (Sigma, C2920) were used as positive and negative controls.

### 4.10 Cytosolic calcium measurements

Fluo‐4, AM (Molecular Probes, F14201) was used according to the manufacturer's instructions to measure mitochondrial and cytosolic Ca^2+^ levels, respectively. HT22 cells were plated at 5x10^3^ cells/well in 96‐well plates in FluoroBrite DMEM and grown overnight. The following day, J147 was added for 6‐8 hr prior to addition of calcium dyes. For calcium ionophore experiments, A23187 (Tocris 1234) and ionomycin (Cayman 10004974) were added at the indicated concentrations along with J147 for 1 hr. Pluronic F‐127 (Molecular Probes, P6866) was used to assist in Fluo‐4 dispersion and used at a final concentration of 0.02%. Fluo‐4 (2.5 μm) was added to cells for a total of 45 min, the first 20 min at RT and the last 25 min at 37°C. Fluorescence was either measured on a Spectramax M5 plate reader or by flow cytometry. FloJo software was used for analysis and results were reported as geometric mean fluorescence intensity.

### 4.11 ATP

ATP measurements were carried out according to the manufacturer's instructions (Molecular Probes A22066). For ATP measurements in drosophila, heads were extracted and homogenized in 6 M guanidine HCl and ATP measurements were carried out according to manufacturer's instructions (Sigma, FLAA).

### 4.12 Transfections

For transfections, 2.5 μg of the H49K‐hIF1 (Laura Formentini et al., 2012) and 2.5 μg GFP‐encoding control plasmid were transfected using Lipofectamine 3000 Reagent (Invitrogen L3000001) for 6‐8 hr and grown overnight. siRNA transfections were carried out using 50 nm ATP5A siRNA and 50 nm control siRNA (Santa Cruz sc‐60228 and sc‐37007) using RNAiMAX reagent (Invitrogen 13778).

### 4.13 Drosophila stocks, culturing conditions, and lifespan analysis

The Canton‐S and w^1118^ lines have been described previously, and F1 offspring from crosses between the two strains (w^1118/+^) were used in this study (Ratliff et al., 2015). Male flies were collected and aged in same‐sex cohorts (25 flies per vial) on standard laboratory media (agar, molasses, yeast, cornmeal, propionic acid, nipagin). Starting at 1 week of age, flies were placed on to standard fly media (control) or vials containing standard media containing 0.1 or 2 μm J147. Flies were maintained at 25°C on a 12‐hr:12‐hr light:dark cycle for the duration of the study. Mortality data were used to generate Kaplan–Meier longevity curves.

### 4.14 Statistical analysis

For the SAMP8 mouse studies, statistical analysis was carried out using one‐way ANOVA followed by Tukey–Kramer multiple comparisons *posts hoc* test. Statistical significance was assessed by analysis of variance (ANOVA) and Student's *t* test where appropriate. A *p* value of <.05 was considered significant. GraphPad Prism 6 was used for statistical analysis.

## AUTHOR CONTRIBUTIONS

J.G., P.M, and D.S. were responsible for the experimental design; M.P. performed the DARTS experiment, and J.G. and D.S. performed J147 pull‐downs, cell culture Western blots, and toxicity studies in MC65 cells; W.F. performed all mass spectrometry analysis. C.C. synthesized J147 and its derivatives; P.M. performed the siRNA experiments in HT22 and MC65 cells, the studies with the AMPK KO MEFs, the ATP synthase activity assay, and the tissue preparation for RNA seq and metabolomic analysis; J.G. was responsible for microscopy, in vitro mitochondrial bioenergetics and toxicity assays, including ATP measurements, Ca^2+^ measurements, as well as remaining survival and toxicity assays; A.C. did the SAMP8 Western blots; M.Pe. was responsible for the transcriptome and metabolome drift analysis; K.F. and E.R. were responsible for the drosophila lifespan study. P. Molto and J. Cueza generously provided the H4K IF1‐overexpression construct; J.G. wrote the manuscript with critical editing by D.S., P.M, and M.Pe.

## CONFLICT OF INTEREST

D.S. is an unpaid advisor for Abrexa Pharmaceuticals, a company working on the development of J147 for AD therapy. The Salk Institute holds the patent for J147.
