# Supplementary material for: The mitochondrial ATP synthase is a shared drug target for aging and dementia
Source: Aging Cell. 2018 Jan 7;17(2):e12715. doi: 10.1111/acel.12715 (PMC5847861; doi:10.1111/acel.12715)
Supplement: Supplementary file 4 [file ACEL-17-e12715-s004.docx]

**Fig. S1. DARTS MS raw data**

Raw data reporting spectral counts of detected proteins preserved after enzymatic digestion. ATP5A was the most enriched protein preserved, with zero spectral counts assigned to the DMSO control, suggesting it to be a putative J147 target.

**Table S1. Pixel colocalization of BJ147 and COXIV**

Percent pixel area overlap of BJ147 and COXIV calculated with FIJI (Schindelin et al. 2012)

**Fig. S2. J147 does not affect the rate of glycolysis in HT22 cells**

Glucose utilization was performed as previously described (Soucek et al. 2003)

**Fig. S3. ATP5A knockdown does not affect levels of other oxphos complexes**

Knockdown of ATP5A in HT22 and MC65 cells has no effect on levels of other oxphos protein complexes.

**Fig. S4. ATP5A knockdown does not alter composition of oxphos complexes.**

NativePAGE gel demonstrates no differences in molecular weights of oxphos complexes in HT22 cells probed with oxphos cocktail and ATP5A antibodies. Statistics are as follows (significance threshold set to .01): *p=.015, p=.001, p=.81, p=.76, p=.27*.

**Fig. S5. J147 increases AMPK phosphorylation in primary rat cortical neurons at 30 min. and 4 hrs**

J147 stimulates AMPK phosphorylation at 100 nM in 7 days of rat primary culture. Cells were treated in Neurobasal media without antioxidants for 30 min. and 4 hrs. and harvested for western blot analysis. *(***p <.001; * p < .05, n=3 per group)*.

**Fig. S6. Drosophila life span analysis raw data**

**References:**

1. Schindelin, J., Arganda-carreras, I., Frise, E., Kaynig, V., Longair, M., Pietzsch, T., . . . Tinevez, J. Y. (2012). Fiji - an open source platform for biological image analysis. *Nature Methods*, *9*(7), 676–682. https://doi.org/10.1038/nmeth.2019.Fiji
2. Soucek, T., Cumming, R., Dargusch, R., Maher, P., Schubert, D., Jolla, L., & Jolla, L. (2003). The regulation of glucose metabolism by HIF-1 mediates a neuroprotective response to amyloid beta peptide the salk institute for biological studies. *Neuron*, *39*, 43–56. https://doi.org/10.1016/S0896-6273(03)00367-2
